# Analyzing life narratives of transsexual people from a developmental point of view

**DOI:** 10.1192/j.eurpsy.2023.238

**Published:** 2023-07-19

**Authors:** J. A. Déri, E. Berán, L. Simon, V. Simon, E. Kovács

**Affiliations:** 1 Department of Psychiatry and Psychotherapy, Division Social Psychiatry, Medical University of Vienna, Vienna, Austria; 2 Department of Psychology, Pázmány Péter University; 3 Department of Psychiatry and Psychotherapy, Semmelweis University, Budapest, Hungary; 4 Department of Psychiatry and Psychotherapy, Bochum University Hospital, Bochum, Germany

## Abstract

**Introduction:**

Transsexuality is a form of gender identity, where persons experience a strong dysphoria or dissatisfaction with the original sexual organs and body they are born with, and believe that they belong to the opposite sex -- so many of such persons undertake sex reassignment surgery. Correlates of transsexuality do not suggest causality but they make it possible to better understand transsexuality and its etiology.

**Objectives:**

It has been shown by previous studies that some signs of transsexuality begin to emerge well before puberty. In our study we wanted to track how transsexual individuals develop from early childhood through adolescence and young adulthood regarding their preferred childhood games and toys, dressing and activities, later sexual partners and social responses to their trans identity.

**Methods:**

59 persons participated as volunteers, who have applied to sex-reassignment surgery and the psychiatric evaluation necessary for the surgery, completed a spontaneously written life narrative as an argument for receiving psychiatric approval for the surgery at the Clinique of Psychiatry and Psychotherapy at Semmelweis University, Budapest, Hungary. Narratives were content-analyzed and coded regarding signs of transsexuality occurring in early childhood, elementary school-age, and puberty, conflict with friends, and family, sexual life, and love-life. All participants have signed the informed –consent form, and agreed to the scientific use of their data. The study has been approved by the local ethical committee.

**Results:**

Our results show that there were 24 female-to male, and 35 male-to female transsexual participants in our sample. 45.7% wanted to dress in clothes of the opposite sex in early childhood, whereas only 1.4% said that he/she did not. 41.4% befriended children who were members of the opposite sex, rather than befriending members of the same sex (4.3%), both (7.1%) or none (4.3%). As a young child 55.7% claimed that they preferred toys and games of the opposite sex, whereas 7.1% claimed they did not. The reaction of their families to signs of transsexuality or unusual behavior was supportive, rejecting, indifferent, or mixed in about equal proportion. In elementary school they befriended members of the opposite sex in 37.1% of the time, whereas they befriended members of the same sex in 1.4% of the time, and did not have friends in 22.9%. In elementary school they preferred activities included those of the opposite sex in 45.7%, and they wanted to dress in clothes of the opposite sex in 54.3% of the cases. In adolescence 10% rejected their biological sex, 40% not merely rejected, but desired the body of the opposite sex, and 25.7% was thinking about body/sex alteration procedures.

**Conclusions:**

We can conclude that signs of gender dysphoria emerge in early childhood, and transsexuality may be characterized and anticipated based on such early signs by parents and care takers.

**Disclosure of Interest:**

None Declared

